# Kimura disease: Unusual presentation in an African American male

**DOI:** 10.1016/j.jdcr.2024.04.022

**Published:** 2024-04-25

**Authors:** Louis J. Born, Kathryn Turney, Juris Germanas

**Affiliations:** a Department of Medicine, Mercy Hospital, Baltimore, Maryland; b Department of Dermatology, University of Maryland Medical Center, Baltimore, Maryland; c Department of Dermatology, Veterans Affairs Medical Center, Baltimore, Maryland

**Keywords:** Kimura disease, reactive lymphadenopathy, subcutaneous

## Introduction

Kimura disease is a rare entity typically involving painless subcutaneous nodules of the head and neck that display characteristic pathologic findings and peripheral blood eosinophilia.^1^ Historically, this disease primarily affects young males of East Asian descent, with a peak incidence in the second and third decade of life.^2^ To create awareness that this condition should be considered when subcutaneous nodules appear in the head and neck region in persons not of Asian descent, we report an unusual case of Kimura disease in an African American male in his 60s.

## Case report

A 64-year-old African American man with no significant prior medical history presented with a mobile, somewhat tender subcutaneous nodule on the left preauricular region that had appeared several years prior. Imaging of the lesion by computed tomography and ultrasound revealed a nonspecific 1 cm subcutaneous mass in the left preauricular region.

**Fig 1:**
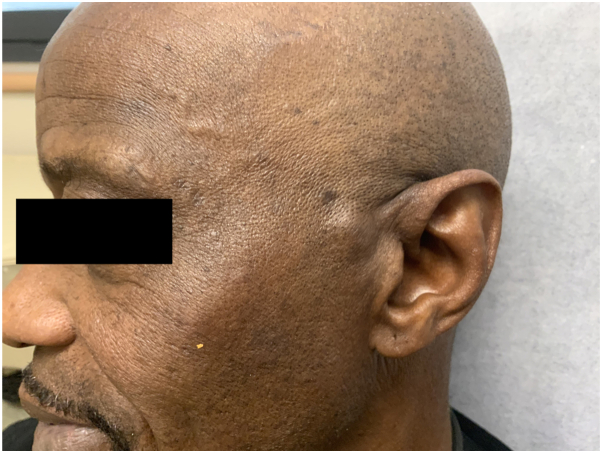
Clinical photograph of nodule on left temple of patient before excision.

Physical examination revealed a solitary, mobile, subcutaneous nodule on the left temple (Fig 1). No overlying punctum or other epidermal changes could be appreciated. The nodule was not pulsatile, nor was it appreciably compressible. A punch biopsy taken into the center of the lesion showed normal-appearing skin structures, and no evidence of a capsule that could suggest a cyst.

In view of the indeterminate diagnostic biopsy, an excision of the lesion was undertaken. Intraoperatively a well-defined 1 cm nodule was revealed that adhered to the superficial temporal artery. In fact, the artery was transected during the resection, requiring ligation.

**Fig 2:**
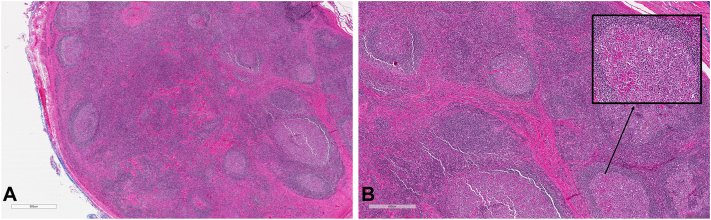
Histopathologic images of specimen from left temple reveal a reactive lymph node with increased eosinophils, foci forming eosinophilic microabscesses. **A**, Hematoxylin-eosin-stained specimen demonstrating lymphoid tissue with hyperplastic follicles and increased capsular and interfollicular fibrosis. **B**, Hematoxylin-eosin-stained high power magnification reveals abundant eosinophils forming an intrafollicular microabscess (insert).

The pathologic specimen was determined to be a lymph node that showed reactive changes. Histomorphologic sections showed lymph node tissue with open sinuses and increased capsular and interfollicular fibrosis (Fig 2, *A*). The lymphoid follicles were enlarged and appeared activated as well. A battery of immunostains for CD20, CD3, bc12, bc16, CD10, CD5, CD30, and ki67 showed no atypical immunophenotypic findings.

Closer examination revealed that the interfollicular regions displayed increased vasculature. Most remarkably, however, were the abundant eosinophils that were observed in the interfollicular as well as intrafollicular areas (Fig 2, *B*). In fact, in several areas, eosinophils aggregated into clusters, termed “microabscesses.” Based on these findings, a diagnosis of Kimura lymphadenopathy was made.

## Discussion

Kimura disease was first described in the Chinese literature in 1937 as “eosinophilic hyperplastic lymphogranuloma” and later coined Kimura disease in 1948 after characterization by Kimura et al^1^ in the Japanese literature. Historically the disease has been reported to primarily affect young Eastern Asian males in the second or third decade of life.^2^^,^^3^ Because of the predominance in young Eastern Asian males, this disease is often not included in the differential diagnosis outside of this demographic. One reported case demonstrated delayed diagnosis of Kimura disease in a young male of African descent due to this very reason.^4^

The most common reported clinical finding of Kimura disease is painless subcutaneous nodules that occur in the head and neck region, with atypical manifestations in the oral cavity.^5^ Patients often display eosinophilia on complete blood count in addition to lymphadenopathy. Rare occurrences of Kimura disease in African males have been previously reported in the epiglottis^6^ and hard palate.^7^ There have also been cases with associated pruritus with lesions of Kimura disease in older patients.^8^ In our case, the patient had associated tenderness with the lesion.

There have also been recent reports analyzing Kimura disease focusing specifically on age. A case series study included 238 patients and found that although Kimura disease was predominately diagnosed with a 17:1 male to female ratio in patients <20 years old, patients diagnosed >40 years of age had a 2:1 male to female ratio. Additionally, time to diagnosis was increased in older patients and clinical presentation involved more cases of associated pruritus compared with younger patients.^8^

In this case, we report an African American male over 60 years of age diagnosed with Kimura disease. The patient presented with a somewhat tender 1 cm subcutaneous nodule of the left temple. Histopathologic sections showed lymph node tissue with open sinuses and increased capsular and interfollicular fibrosis. Abundant eosinophils were also present in interfollicular areas forming microabscesses, leading to the diagnosis of Kimura disease. Elevated eosinophils were in fact observed initially on the patient’s complete blood count, but returned to normal levels after removal of the lesion.

It is important to continue to follow the clinical course of all patients diagnosed with Kimura disease. Clinical presentation, imaging, and histologic analysis can closely resemble malignancy, such as Hodgkin lymphoma.^9^ One reported case involving an African male who was initially diagnosed with Kimura disease had progression of the disease with repeat biopsy being diagnostic of Hodgkin lymphoma.^2^ In another instance, positron emission tomography-computed tomography showed uptake of fluorodeoxyglucose suggesting Hodkin lymphoma, but a final diagnosis of Kimura disease was made on histologic analysis. Another case involving a White woman with a nontender nodule of the neck primarily included malignancy on the differential, but was eventually diagnosed with Kimura disease.^10^

To our knowledge, this is the first reported case of Kimura disease in an African American male over the age of 60 years. Although the patient had no renal deficiencies, he did have peripheral blood eosinophilia, a common finding in Kimura disease, that in fact subsided after removal of the lymph node. The other reasonable diagnosis, angiolymphoid hyperplasia with eosinophilia, was ruled out by the presence of eosinophilic microabscesses, as well as the absence of characteristic thick-walled vessels with plump endothelial cells in the pathology. This case highlights the importance of including Kimura disease as a differential in all cases involving subcutaneous nodules of the head and neck region regardless of age or race. As there have been reported cases involving nontraditional presentation and misdiagnosis of the disease, it is important to be aware of the variable clinical presentations and the need to follow up disease progression.

## Conflicts of interest

None disclosed.
